# A Rare Case of *Raoultella planticola* and *Enterococcus casseliflavus* Coinfection

**DOI:** 10.1155/2022/3377331

**Published:** 2022-05-23

**Authors:** Varsha Prasad, Baina Barouni, Bashar Khiatah, Musab Saeed

**Affiliations:** ^1^Community Memorial Health Systems, Department of Internal Medicine, Ventura, CA, USA; ^2^Western University, School of Medicine, Ventura, CA, USA; ^3^Community Memorial Health Systems, Department of Infectious Disease, Ventura, CA, USA

## Abstract

*Raoultella planticola*, a Gram-negative bacterium, is a nonmotile rod usually found in soil and aquatic environments. It can be found in association with gastrointestinal malignancy. *Enterococcus casseliflavus* is a rare vancomycin-resistant *Enterococcus* that is responsible for some bacteremia. Our case describes a unique presentation of colonization with both *R. planticola* and *E. casseliflavus* isolated from the biliary stent isolates of a patient with known pancreatic malignancy and concomitant *E. casseliflavus* bacteremia. This is the first case ever reported of infection with both species.

## 1. Introduction

This case report describes a patient with pancreatic malignancy presenting for ascending cholangitis with polymicrobial infection including *E. casseliflavus* bacteremia and *E. casseliflavus* and *R. planticola* isolated from bile acid. *R. planticola* is an encapsulated, nonmotile, aerobic Gram-negative rod that rarely causes infections in humans [1]. There may be more *R. planticola* infections than reported, since previous studies have shown that *R. planticola* may be confused for *Klebsiella* species upon isolation [2]. *E. casseliflavus* is a motile member of the *Enterococcus* species, known to commonly cause urinary tract infections, intraabdominal sepsis, and surgical wound infections [3]. VanC gene cluster is an intrinsic part of *E. casseliflavus* and provides the bacteria with resistance to vancomycin [4]. To our knowledge, this is the first case demonstrating coinfection of both *E. casseliflavus* and *R. planticola*. This case also demonstrates a possible association between *R. planticola* and gastrointestinal malignancy.

## 2. Case Description

A 74-year-old male with pancreatic adenocarcinoma presented with abdominal pain, fevers, and emesis. He recently presented two weeks prior for similar symptoms at which time a common bile duct stent was placed. His pain was located to the right upper quadrant, characterized as a sharp pain, and worsened with food intake. Fevers measured up to 38.5°C at home. Admission labs significant for white blood cell count of 4.6 K/uL (normal 4.8–10.8 K/uL). Computed tomography imaging revealed an irregular pancreatic head mass and associated pancreatic ductal dilation and increased peripancreatic inflammatory change when compared to prior images. There was concern for acute pancreatitis due to recent common bile duct stent placement or worsening pancreatic ductal obstruction. Antibiotic regimen with piperacillin-tazobactam was initiated.

**Figure 1:**
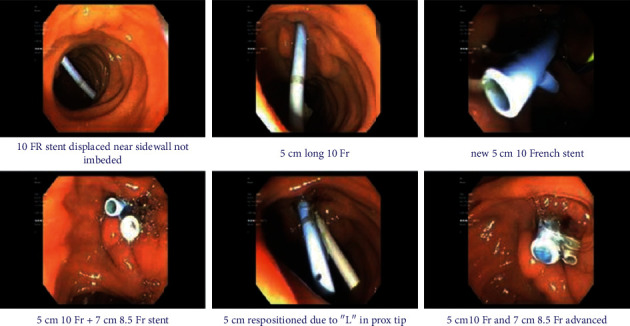
ERCP images showing (in order) stent removal and replacement with a new 5 cm 10 French stent and 7 cm 8.5 French stent.

Blood cultures were collected from peripheral blood samples on admission. On hospital day one, blood cultures revealed growth of *E. casseliflavus* in two out of two bottles via the VITEK MS automated mass spectrometry microbial identification system. The patient underwent endoscopic retrograde cholangiopancreatography with stent removal and exchange with bile cultures obtained (Figure 1). Local bile cultures from the stent site resulted positive for *Enterobacter cloacae*, *E. casseliflavus*, *R. planticola*, and *Candida albicans*. *E. casseliflavus* and *R. planticola* were isolated in thioglycolate broth. His antibiotic regimen was adjusted accordingly to cefepime, daptomycin, oral metronidazole, and oral fluconazole. His symptoms improved postoperatively and laboratory values stabilized. The patient was instructed to continue his antibiotic regimen for a total of two weeks of treatment and to undergo weekly laboratory monitoring with complete blood count, comprehensive metabolic panel, and creatine phosphokinase.

He was seen in outpatient follow-up one month following discharge. Upon completion of his antibiotic course, his symptoms resolved and he no longer had fevers, abdominal pain, or emesis. His lab values remained stable without increase in inflammatory markers.

## 3. Discussion

This case demonstrates a rare case of polymicrobial infection involving both *R. planticola* and *E. casseliflavus* isolated from the bile of a 74-year-old male patient with pancreatic cancer. Extensive review of the literature has not, to our knowledge, revealed a similar case of polymicrobial infection. Our case also demonstrates *R. planticola* infection associated in the setting of pancreatic cancer, which has been rarely described in the literature.

*R. planticola* is a commensal Gram-negative aerobic rod bacteria closely related to the *Klebsiella* species. Found in water and soil, it is rarely a cause of serious infection in humans. Case reports demonstrate *R. planticola* bacteremia in the setting of gastric malignancy in an otherwise asymptomatic patient [5]. This is relevant in our patient's case, given our patient's history of pancreatic cancer. In a literature review by Yamamoto, *R. planticola* was found in 70.6% of patients. The malignancies included biliary tract neoplasms (29.2%) and pancreatic neoplasms (16.7%) [5]. Interestingly, 83.3% of these patients with malignancies were treated with chemotherapy or stem cell transplant before the development of bacteremia, suggesting an immunocompromised state, either related to an underlying malignancy or associated chemotherapy, was associated with development of *R. planticola* bacteremia [5]. In our case, *R. planticola* was isolated from bile following CBD stent placement. *R. planticola* has been found to cause cholangitis [6] and cholecystitis [7]. *R. planticola* may be an underestimated cause of severe infection and should be suspected in patients with a history of cancer and recent invasive medical procedures [6]. The true incidence of *R*. *planticola* infections or coinfections may be underestimated due to difficult cultivation [6]. Literature review by Salmaggi shows 12 previously reported cases of *R. planticola* infection occurred in mainly males (81.8%), associated frequently with neoplasm (30.8%) and recent trauma or invasive procedures (53.8%). Another case report by Teo describes the first report of biliary sepsis with *R. planticola*. One possible theory is that *R. planticola* natural course of infection occurs when systemic impairment of the host immune system enables dormant colonizers to become invasive [6].

Another case report by Yokota describes an immunocompromised patient with metastatic neck cancer who developed infection with *R. planticola* after undergoing ERCP. The patient subsequently went on to develop cholangitis with septic shock; presumably, the patient was colonized with *R. planticola* in the GI tract before ERCP [8]. Another case report describes *R. planticola* bacteremia following consumption of seafood in a patient undergoing chemotherapy, proton pump inhibitor use, and with cholangitis [9].

*E. casseliflavus* is a yellow, motile member of the *Enterococcus* species and known to have intrinsic low level vancomycin resistance characteristic of *E. casseliflavus* [10]. Enterococci are part of normal gut flora and intrinsically resistant to beta-lactam agents and aminoglycosides and were the first bacteria to acquire vancomycin resistance. Therapy with cephalosporins and vancomycin, among other antimicrobial agents, may play a role in increasing colonization with these organisms. The most common enterococci infection is urinary tract infection. It can cause invasive infections in immunocompromised patients such as hematologic malignancy, renal failure, diabetes mellitus, bone marrow transplant, antithrombin III deficiency, astrocytoma, chronic osteomyelitis, and organ transplant recipient [10, 11]. *E. casseliflavus* is a rare pathogen, but must be considered in at-risk patients to assist in antibiotic selection. Note vancomycin therapy is not recommended for VanC VRE infection, even for strains that are susceptible in vitro [11]. *E. casseliflavus* is frequently associated with polymicrobial bacteremia and biliary tract disease [11].

In a case series by Choi, of 56 patients with *E. casseliflavus* bacteremia, the most common portal of entry was biliary disease (76.8%), in which adequate drainage frequently relieved the septic conditions. *E. casseliflavus* is commonly associated with biliary tract disease [11]. *E. casseliflavus* is also an uncommon but important agent involved in SBP and bacterascites [12]. Another case report describes a patient with significant underlying conditions and a case of “spontaneous” enterococcal meningitis, of which *E. casseliflavus* was isolated [13].

## 4. Conclusion

A review of the literature shows *R. planticola* and *E. casseliflavus* as independent isolates in bacteremia and/or cholangitis, but never seen together. This case also demonstrates how *R. planticola* may have an association with underlying malignancy.

## Data Availability

The data used to support the findings of this study are included within the article.
